# Neuroprotective and Anti-Inflammatory Effects of a Hydrophilic Saffron Extract in a Model of Glaucoma

**DOI:** 10.3390/ijms20174110

**Published:** 2019-08-22

**Authors:** Jose A. Fernández-Albarral, Ana I. Ramírez, Rosa de Hoz, Nerea López-Villarín, Elena Salobrar-García, Inés López-Cuenca, Ester Licastro, Antonio M. Inarejos-García, Paula Almodóvar, Maria D. Pinazo-Durán, José M. Ramírez, Juan J. Salazar

**Affiliations:** 1 Instituto de Investigaciones Oftalmológicas Ramón Castroviejo, UCM, 28040 Madrid, Spain; 2 Departamento de Inmunología, Oftalmología y ORL, Facultad de Óptica y Optometría, UCM, 28037 Madrid, Spain; 3 Pharmactive Biotech Products, SL. Parque Científico de Madrid, Faraday 7, 28049 Madrid, Spain; 4 Unidad de Investigación Oftalmológica Santiago Grisolia, Universidad de Valencia, 46017 Valencia, Spain; 5 Departamento de Inmunología, Oftalmología y ORL, Facultad de Medicina, UCM, 28040 Madrid, Spain

**Keywords:** ganglion cells, microglia, retina, experimental glaucoma, saffron extract, Brn3a, Iba-1, P2RY12, neuroprotection, neuroinflammation

## Abstract

Glaucoma is a neurodegenerative disease characterized by the loss of retinal ganglion cells (RGCs). An increase in the intraocular pressure is the principal risk factor for such loss, but controlling this pressure does not always prevent glaucomatous damage. Activation of immune cells resident in the retina (microglia) may contribute to RGC death. Thus, a substance with anti-inflammatory activity may protect against RGC degeneration. This study investigated the neuroprotective and anti-inflammatory effects of a hydrophilic saffron extract standardized to 3% crocin content in a mouse model of unilateral, laser-induced ocular hypertension (OHT). Treatment with saffron extract decreased microglion numbers and morphological signs of their activation, including soma size and process retraction, both in OHT and in contralateral eyes. Saffron extract treatment also partially reversed OHT-induced down-regulation of P2RY12. In addition, the extract prevented retinal ganglion cell death in OHT eyes. Oral administration of saffron extract was able to decrease the neuroinflammation associated with increased intraocular pressure, preventing retinal ganglion cell death. Our findings indicate that saffron extract may exert a protective effect in glaucomatous pathology.

## 1. Introduction

Glaucoma is one of the leading causes of irreversible blindness in the world. It is characterized by retinal ganglion cell (RGC) death which leads to a progressive visual field loss and blindness [1]. Intraocular pressure (IOP) is the only modifiable glaucoma risk factor, and it is the target of the majority of currently used treatments. However, IOP control does not always prevent glaucomatous neurodegeneration, which is irreversible [2]. Therefore, early diagnosis of neurodegeneration, understanding of its pathogenic mechanisms, and the development of new neuroprotective therapies represent major challenges for treating this disease.

To study RGC death in glaucoma, a variety of rat and mouse models have been used, including the use of hypertonic saline injection into the episcleral veins, cauterization or ligation of episcleral veins, or laser photocoagulation of the perilimbal region [3,4]. The studies performed in these models have proposed various mechanisms of RGC death in glaucoma, including glutamate excitotoxicity, aggregation of misfolded proteins, mitochondrial dysfunction, oxidative stress, neurotrophic deprivation, and neuroinflammation, among others [2,5,6,7].

Microglial activation is a hallmark of neuroinflammation in the central nervous system. The microglia, as immunoregulatory resident cells, may be able to initiate the immune response during glaucomatous neurodegeneration [8,9]. Microglial activation has been associated with RGC loss, both in human and in experimental models of glaucoma [6,10,11,12,13], and its inhibition reduces RGC death [14,15]. Signs of microglial activation were observed in a mouse model of unilateral, laser-induced ocular hypertension (OHT) at different time points after laser induction [6,7,10,11]. These signs, which have been observed both in eyes with OHT and in their contralateral normotensive eyes, include an increase in microglial cell number and soma size as well as process retraction. The presence of early microglial activation in both eyes at 24 h after laser exposure implicates the immune system in OHT-related neurodegeneration [10].

The related literature suggests that treatments with neuroprotective, antioxidant, and anti-inflammatory characteristics could reduce microglial activation and prevent RGC death induced by OHT.

Saffron (dried stigmas of *Crocus sativus* L.) is a spice that has been used since ancient times in food preparation [16]. In addition to its organoleptic attributes, saffron is reported to have important antioxidant, anti-inflammatory, and anti-apoptotic activities [17,18,19,20]. These biological activities arise from its bioactive constituents. Stigmas contain many more than 100 metabolites, including carotenoids, crocin isomers, and diverse additional compounds [21,22,23], which may have several pharmacological and therapeutic applications [24]. Crocin isomers are transformed into crocetin during digestion in animals and humans [25,26]. Crocetin can be distributed in different tissues because of the weak interaction between crocetin and albumin [26]. An in vitro study showed that crocetin can penetrate the blood–brain barrier (BBB) and reach the central nervous system by passive transcellular diffusion [27]. Furthermore, an in vivo experiment demonstrated that trans-crocin 4 (TC4 has been found to be the most abundant crocin among all crocin types) is able to cross the BBB and build up levels in the mouse brain [28]. However, an experiment done in albino rats treated with saffron showed that in healthy retina, there were no metabolites related to saffron, but in damaged retinas (due to high intensity light exposure), crocin could reach the retinal tissue following damage to the blood retinal barrier [29].

Increasing evidence from both experimental models [30,31,32,33] and clinical studies in patients [30,34] supports the neuroprotective effect of saffron components in neurodegenerative conditions such as Alzheimer’s and Parkinson’s diseases, In addition, the saffron extracts (crocin and crocetin) can decrease neuroinflammation by reducing the production of varius neurotoxic molecules from activated microglia (nitric oxide, tumor necrosis factor (TNF)-α, interleukin (IL)-1β, and reactive oxygen species (ROS)), as demonstrated in animal models of lipopolysaccharide (LPS)-induced inflammation [35,36]. Thus, these compounds may exert neuroprotective effects in the central nervous system.

In the eye, beneficial effects of saffron extracts have been demonstrated in different animal models of retinal damage. Crocin significantly increases the blood flow in the retina and choroid, and presumably improves oxygenation and the nutrient supply in retinal structures after an acute increase in IOP in rabbits [37]. Crocetin was shown to prevent ischemia-induced retinal damage through oxidative stress reduction in a mouse model of ischemia/reperfusion [38]. In addition, crocetin was shown to suppress caspase-3 and caspase-9 activities, thereby protecting the retina against neuronal damage, in both an in vitro model (retinal damage induced by tunicamycin and H_2_O_2_) and an in vivo model (light-induced photoreceptor degeneration in mice) [18]. Studies have even investigated the therapeutic potential of these compounds in animal models of neurodegenerative ocular diseases such as aged-related macular degeneration (AMD) [29], diabetic retinopathy (DR) [39], and retinitis pigmentosa [40,41]. This beneficial effect also has been observed in patients with AMD [42,43] and diabetic maculopathy [44]. However, studies focusing on glaucoma are scarce, and we are aware of only one publication demonstrating that oral saffron supplementation can significantly reduce IOP in patients with primary open angle glaucoma [45].

In the present work, we aimed to analyze whether a natural saffron extract standardized to 3% crocin content could repress retinal microglial activation and prevent RGC death in a mouse model of unilateral, laser-induced OHT.

## 2. Results

### 2.1. Intraocular Pressure

**Figure 1:**
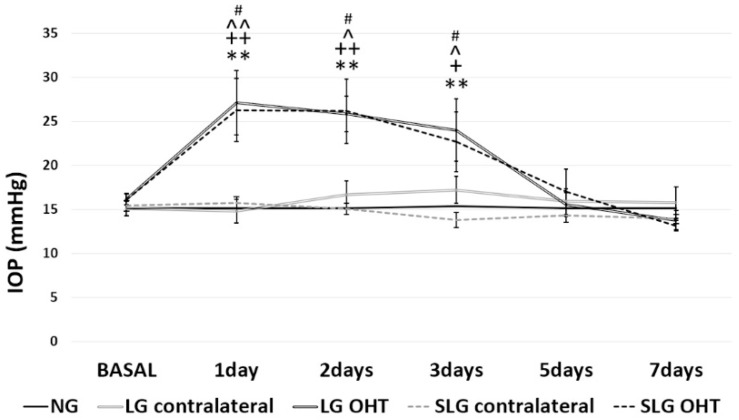
Intraocular pressure (IOP) in the different study groups after laser treatment. Data are shown as means (± standard deviation, SD). Abbreviations: Naïve age-matched control (NG), lasered group contralateral (LG contralateral), lasered group ocular hypertension (LG OHT), lasered group contralateral + saffron (SLG contralateral), lasered group ocular hypertension + saffron (SLG OHT). Statistical significance indicators: ** *p* < 0.01 for NG vs. LG OHT; ^+^ *p* < 0.05, ^++^ *p* < 0.01 for NG vs. SLG OHT; ^^^ *p* < 0.05, ^^^^ *p* < 0.01 LG contralateral vs. LG OHT; ^#^ *p* < 0.05 SLG contralateral vs. SLG OHT.

IOP in lasered group (LG) OHT eyes differed significantly from that in naïve eyes on days 1–3 after treatment (all *p* < 0.01), and from the contralateral eyes on day 1 (*p* < 0.01) as well as days 2 and 3 (*p* < 0.05). In saffron + lasered group (SLG) animals, OHT eyes showed significantly higher IOP values than naïve eyes on days 1 and 2 (*p* < 0.01) and day 3 (*p* < 0.05), and from contralateral eyes on days 1–3 (all *p* < 0.05). On day 5, IOP began to fall in LG OHT and SLG OHT eyes, becoming similar to naïve values by day 7 (Figure 1).

### 2.2. Saffron Extract Did Not Produce Changes in Retinal Tissue in Naïve Eyes

Before studying the effects of saffron extract on the RGC number and morphological signs of microglial activation after OHT laser induction, we first compared NG and SCG eyes by counting the RGC number and analyzing Iba-1 + cells characteristics (cell number, arbor area, soma size, and vertical processes connecting OPL (outer plexiform layer) and OS (photoreceptor outer segment layer). The analysis of these parameters did not show significant differences between the two groups.

### 2.3. Saffron Extract Protected RGCs (Retinal Ganglion Cells) Against Damage Produced by Laser-Induced OHT (Ocular Hypertension)

**Figure 2:**
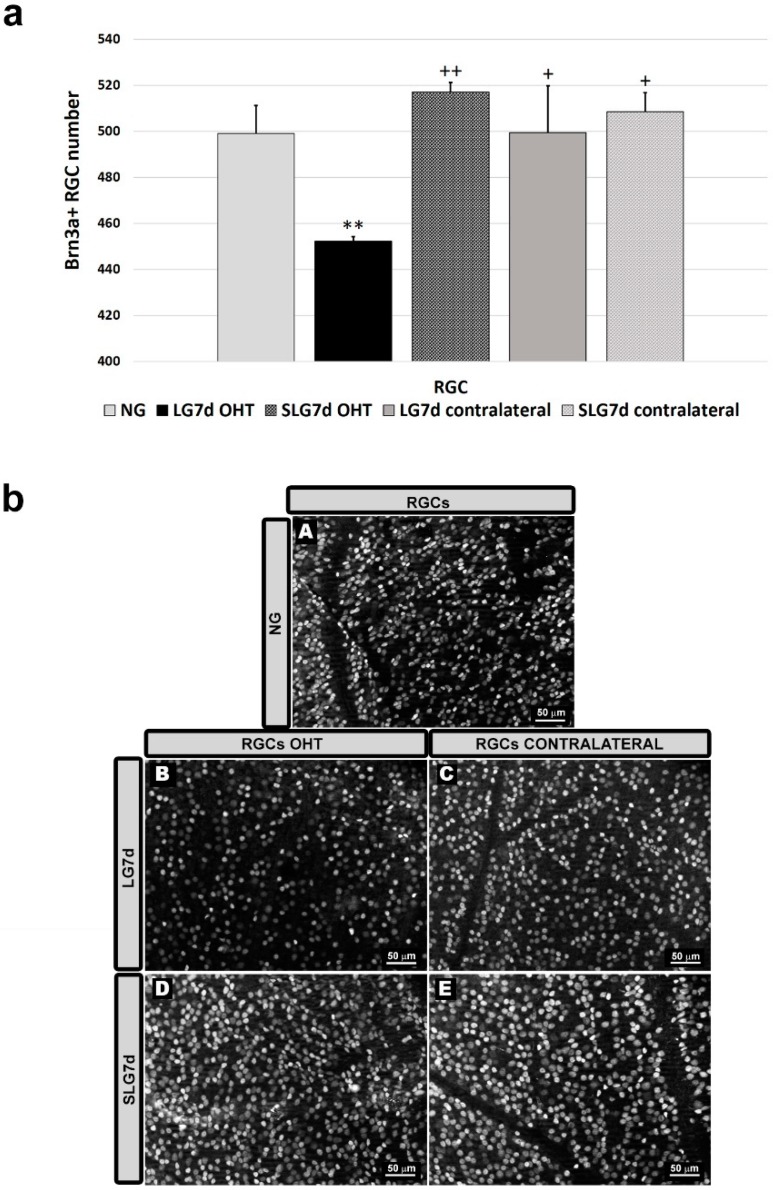
Comparison of numbers of Brn3a + retinal ganglion cells (RGCs) in eyes untreated and treated with saffron extract at day 7 after ocular hypertension (OHT) induction. (**a**) Histograms showing the mean number (± standard deviation, SD) of Brn3a + cells per area of 0.1502 mm^2^. (**b**) Immunohistochemical images of anti-Brn3a stained retinal whole mounts. (**A**) NG, (**B**) LG7d OHT, (**C**) LG7d contralateral, (**D**) SLG7d OHT eyes, and (**E**) SLG7d contralateral. Abbreviations: Naïve age-matched control (NG), lasered group contralateral (LG7d contralateral), lasered group ocular hypertension (LG7d OHT), lasered group contralateral + saffron (SLG7d contralateral), lasered group ocular hypertension + saffron (SLG7d OHT). Statistical significance indicators: ** *p* < 0.01 vs. NG; ^+^ *p* < 0.05, ^++^ *p* < 0.01 vs. LG7d OHT.

Lasered group ocular hypertension (LG7d OHT) was associated with a significant reduction in the number of Brn3a + RGCs compared with NG (*p* < 0.01) and with LG7d contralateral eyes (*p* < 0.05). However, saffron extract treatment counteracted this reduction, and there was a significant difference between lasered group ocular hypertension + saffron (SLG7d OHT) and LG7d OHT (*p* < 0.01). No significant differences were found between contralateral eyes in LG7d and SLG7d (Figure 2).

**Figure 3:**
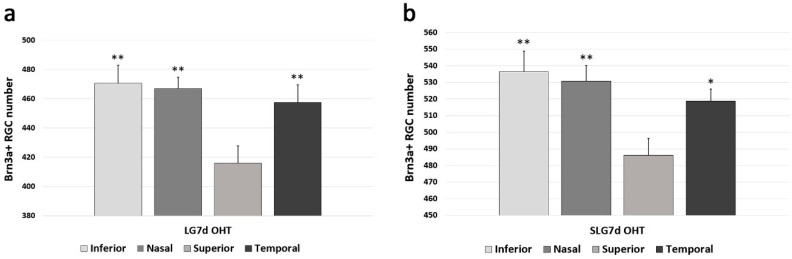
The number of Brn3a + RGCs in eyes untreated and treated with saffron extract at day 7 after ocular hypertension (OHT) induction in the different retinal zones (inferior, nasal, superior, and temporal): (**a**) Brn3a + RGCs number in LG7d OHT; (**b**) Brn3a + RGCs number in SLG7d OHT. Histograms show the mean number (± standard deviation, SD) of Brn3a + RGCs per area of 0.1502 mm^2^. Abbreviations: Lasered group ocular hypertension (LG7d OHT), lasered group ocular hypertension + saffron (SLG7d OHT). Statistical significance indicators: * *p* < 0.05, ** *p* < 0.01 vs. superior.

In LG7d OHT eyes, the comparison of the Brn3a + RGCs count between different retinal zones showed that, in the superior area, the number of Brn3a + RGCs was significantly lower with respect to the inferior, nasal, and temporal areas (all *p* < 0.01) (Figure 3a). In SLG7d OHT eyes, the number of Brn3a + RGCs in the superior area was smaller than in inferior, nasal (*p* < 0.01), and temporal (*p* < 0.05) areas (Figure 3b). In LG7d OHT eyes, the number of Brn3a RGCs in the superior, inferior, nasal, and temporal zones was lower, in a similar proportion, than in SLG7d OHT eyes.

### 2.4. Saffron Extract Reduced the Morphological Signs of Microglial Activation Produced by Laser-Induced OHT

#### 2.4.1. Iba-1 + Cell Number in the OS (Photoreceptor Outer Segment Layer), OPL (Outer Plexiform Layer), and IPL (Inner Plexiform Layer)

**Figure 4:**
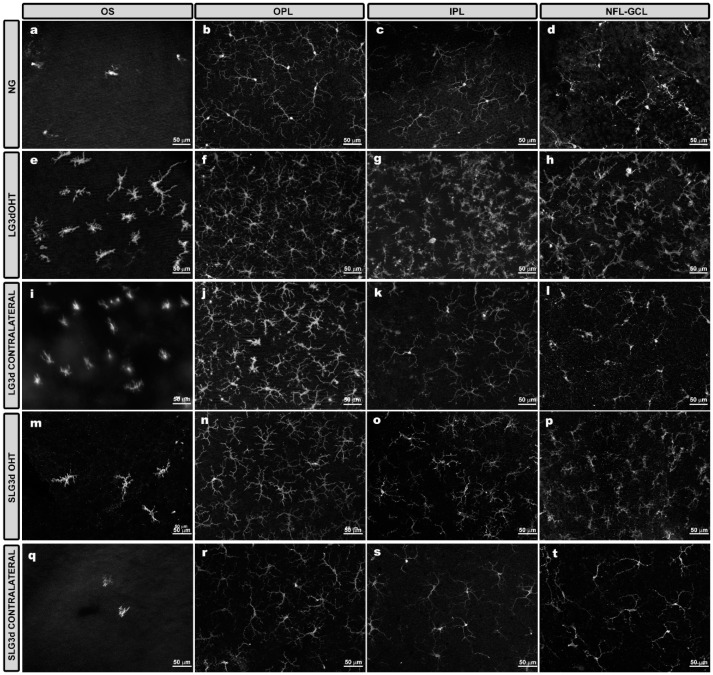
Comparison of Iba-1 + cells in eyes untreated and treated with saffron extract in the photoreceptor outer segment layer (OS), outer plexiform layer (OPL), inner plexiform layer (IPL), and the nerve fiber—ganglion cell layer (NFL-GCL) at day 3 after ocular hypertension (OHT) induction. The images show retinal whole mounts of Iba-1 immunostaining. (**a**–**d**) NG; (**e**–**h)** LG3d OHT; (**i**–**l**) LG3d contralateral; (**m**–**p**) SLG3d OHT; (**q**–**t**) SLG3d contralateral. (**a**,**e**,**i**,**m**,**q**) OS; (**b**,**f**,**j**,**n**,**r**) OPL; (**c**,**g**,**k**,**o**,**s**) IPL; (**d**,**h**,**l**,**p**,**t**) NFL-GCL. Abbreviations: Naïve age-matched control (NG), lasered group contralateral (LG3d contralateral), lasered group ocular hypertension (LG3d OHT), lasered group contralateral + saffron (SLG3d contralateral), lasered group ocular hypertension + saffron (SLG3d OHT).

**Figure 5:**
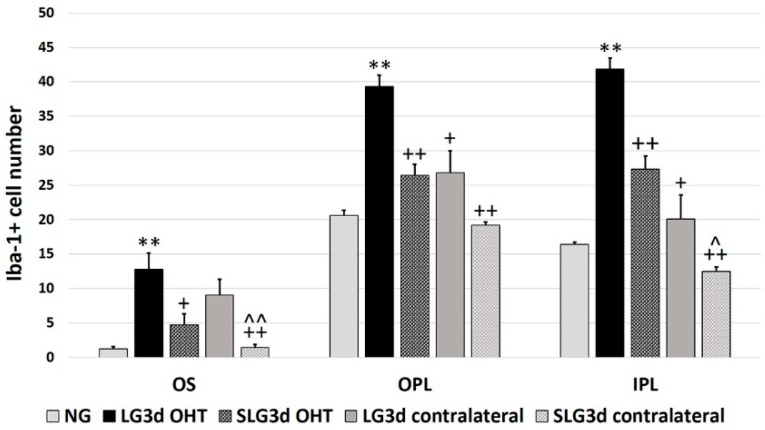
Number of Iba-1 + cells at day 3 after ocular hypertension (OHT) induction in the photoreceptor outer segment layer (OS), outer plexiform layer (OPL), and inner plexiform layer (IPL). Histograms show the mean number (± standard deviation, SD) of Iba-1 + cells per area of 0.1502 mm^2^. Abbreviations: Naïve age-matched control (NG), lasered group contralateral (LG3d contralateral), lasered group ocular hypertension (LG3d OHT), lasered group contralateral + saffron (SLG3d contralateral), lasered group ocular hypertension + saffron (SLG3d OHT). Statistical significance indicators: ** *p* < 0.01; vs. NG. ^+^ *p* < 0.05, ^++^ *p* < 0.01; vs. LG3d OHT.^^^ *p* < 0.05, ^^^^ *p* < 0.01; vs. LG3d contralateral.

The number of Iba-1 + cells was significantly higher in LG3d OHT eyes than in NG eyes in the OS, OPL, and IPL (all *p* < 0.01), and higher in LG3d OHT eyes than in LG3d contralateral eyes in the OPL and IPL (both *p* < 0.05). The OHT-induced increase in Iba-1 + cell number was smaller in the eyes treated with saffron extract (SLG3d OHT) than in untreated eyes (LG3d OHT) (*p* < 0.05 in the OS, and *p* < 0.01 in the OPL and IPL). In SLG3d contralateral eyes, the number of Iba-1 + cells was smaller than in LG3d contralateral eyes (*p* < 0.01 in the OS and *p* < 0.05 in the IPL) (Figure 4 and Figure 5).

In OHT eyes, no differences were found in the number of Iba-1 + cells in the OS, OPL, and IPL among the superior, inferior, nasal, and temporal retinal zones in the LG3d OHT eyes and SLG3d OHT eyes.

#### 2.4.2. Area of the Retina Occupied by Iba-1 + Cells (Iba1-RA) in the NFL-GCL

**Figure 6:**
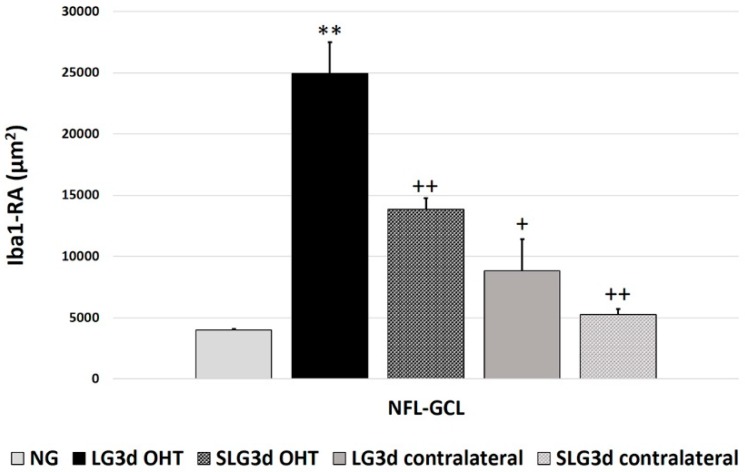
Area of the retina occupied by Iba-1 + cells (Iba1-RA) in eyes untreated and treated with saffron extract at day 3 after ocular hypertension (OHT) induction in the the nerve fiber—ganglion cell layer (NFL-GCL). Histograms show the mean (± standard deviation, SD) of Iba1-RA per area of 0.1502 mm^2^. Abbreviations: Naïve age-matched control (NG), lasered group contralateral (LG3d contralateral), lasered group ocular hypertension (LG3d OHT), lasered group contralateral + saffron (SLG3d contralateral), lasered group ocular hypertension + saffron (SLG3d OHT). Statistical significance indicators: ** *p* < 0.01; vs. NG. ^+^ *p* < 0.05, ^++^ *p* < 0.01; vs. LG3d OHT.

In the NFL-GCL, the Iba1-RA was significantly higher in LG3d OHT eyes than in NG eyes (*p* < 0.01) and LG3d contralateral eyes (*p* < 0.05). Saffron-treated eyes (SLG3d OHT) showed significantly smaller Iba1-RA values than LG3d OHT eyes (*p* < 0.01). No significant differences were found between contralateral eyes in LG3d and SLG3d (Figure 4 and Figure 6).

In OHT eyes, no differences were found in Iba1-RA among the superior, inferior, nasal, and temporal retinal zones in the LG3d OHT eyes and SLG3d OHT eyes.

#### 2.4.3. Arbor Area of Iba-1 + Cells in the OPL and IPL

**Figure 7:**
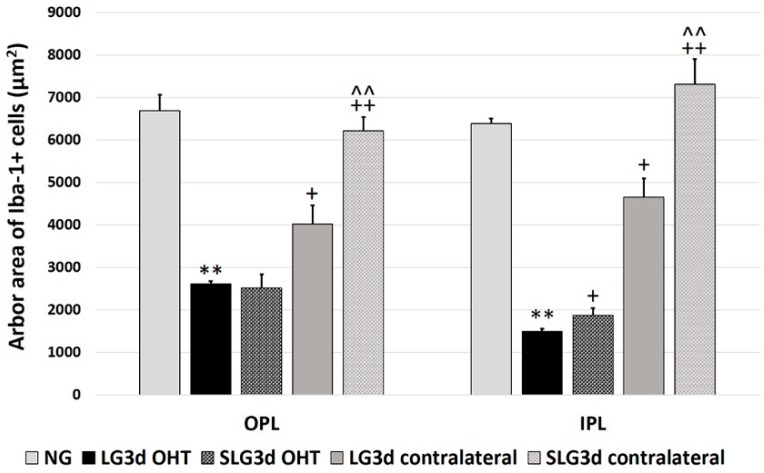
Comparison of the arbor area of Iba-1 + cells in the outer plexiform layer (OPL) and inner plexiform layer (IPL) in eyes untreated and treated with saffron extract at 3 days after ocular hypertension (OHT) induction. Histograms show the mean arbor area (± standard deviation, SD) of Iba-1 + cells. Abbreviations: Naïve age-matched control (NG), lasered group contralateral (LG3d contralateral), lasered group ocular hypertension (LG3d OHT), lasered group contralateral + saffron (SLG3d contralateral), lasered group ocular hypertension + saffron (SLG3d OHT). Statistical significance indicators: ** *p* < 0.01; vs. NG. ^+^ *p* < 0.05, ^++^ *p* < 0.01; vs. LG3d OHT. ^^^^ *p* < 0.01; vs. LG3d contralateral.

The arbor area of Iba-1 + cells was significantly smaller in LG3d OHT eyes than in NG eyes (*p* < 0.01 in both the OPL and IPL) and LG3d contralateral eyes (*p* < 0.05 in both the OPL and IPL), indicating the retraction of processes. The arbor area was significantly greater in SLG3d OHT than in LG3d OHT in the IPL only (*p* < 0.05). The arbor area of Iba-1 + cells was significantly greater in the SLG3d contralateral eyes than in the LG3d contralateral eyes (*p* < 0.01 in both the OPL and IPL) (Figure 4 and Figure 7).

#### 2.4.4. Cell Body area of Iba-1 + Cells in the OPL, IPL, and NFL-GCL

**Figure 8:**
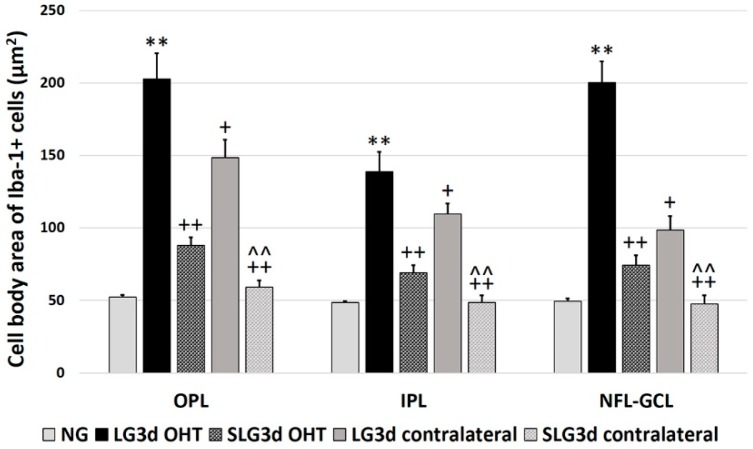
Cell body area of Iba-1 + cells in the outer plexiform layer (OPL), inner plexiform layer (IPL), and nerve fiber—ganglion cell layer (NFL-GCL) in eyes untreated and treated with saffron extract at day 3 after ocular hypertension (OHT) induction. Histograms show the mean cell body area (± standard deviation, SD) of Iba-1 + cells. Abbreviations: Naïve age-matched control (NG), lasered group contralateral (LG3d contralateral), lasered group ocular hypertension (LG3d OHT), lasered group contralateral + saffron (SLG3d contralateral), lasered group ocular hypertension + saffron (SLG3d OHT). Statistical significance indicators: ** *p* < 0.01; vs. NG. ^+^ *p* < 0.05, ^++^ *p* < 0.01 vs. LG3d OHT. ^^^^ *p* < 0.01; vs. LG3d contralateral.

Iba-1 + cells in the OPL, IPL, and NFL-GCL of LG3d OHT eyes showed significantly larger cell body areas than in NG eyes and LG3d contralateral eyes (all *p* < 0.01). Treatment with saffron extract significantly reduced the cell body area in SLG3d OHT eyes when compared with the untreated group (LG3d OHT) (*p* < 0.01 in the OPL, IPL, and NFL-GCL). A decrease in the soma size was also observed in SLG3d contralateral eyes compared with LG3d contralateral eyes (*p* < 0.01 in all layers analyzed) (Figure 4 and Figure 8).

#### 2.4.5. Iba-1 + Vertical Processes

**Figure 9:**
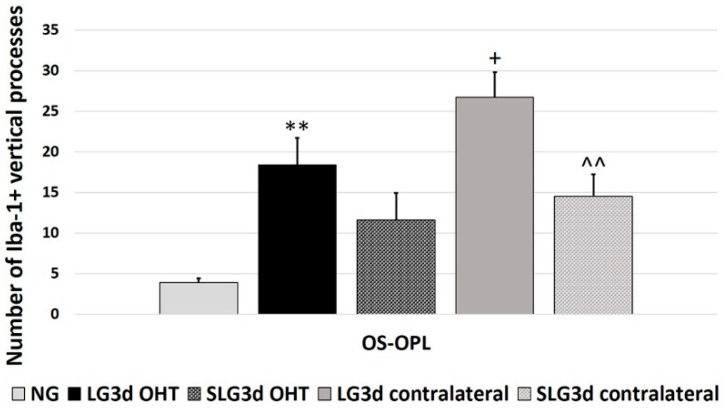
Comparison of Iba-1 + vertical processes (VP) in eyes untreated and treated with saffron extract at 3 days after ocular hypertension (OHT) induction. Histograms show the mean number (± standard deviation, SD) of Iba-1 + vertical processes. Abbreviations: Naïve age-matched control (NG), lasered group contralateral (LG3d contralateral), lasered group ocular hypertension (LG3d OHT), lasered group contralateral + saffron (SLG3d contralateral), lasered group ocular hypertension + saffron (SLG3d OHT). Statistical significance indicators: ** *p* < 0.01; vs. NG. ^+^ *p* < 0.05; vs. LG3d OHT. ^^^^ *p* < 0.01; vs. LG3d contralateral.

The number of vertical processes connecting OPL and OS significantly increased in LG3d OHT eyes compared with NG eyes (*p* < 0.01). However, this number was greater in LG3d contralateral eyes than in LG3d OHT eyes (*p* < 0.05). No significant differences were found between treated and untreated OHT eyes. Contralateral eyes treated with saffron extract showed a decrease in the number of vertical processes in comparison with untreated ones (*p* < 0.01) (Figure 9).

### 2.5. Saffron Extract Prevented Down-Regulation of Microglial P2RY12 Expression in OHT Eyes

**Figure 10:**
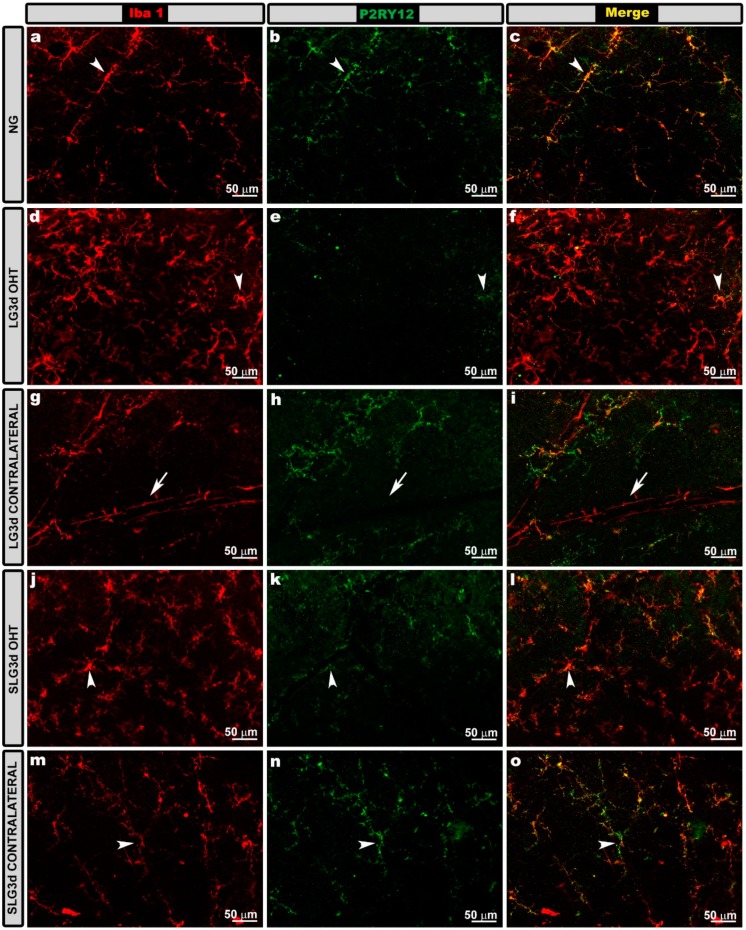
P2RY12 expression in the nerve fiber—ganglion cell layer (NFL-GCL) in eyes untreated and treated with saffron extract at day 3 after ocular hypertension (OHT) induction. Retinal whole mounts with double immunostaining against Iba-1 (**a**,**d**,**g**,**j**,**m**) and P2RY12 (**b**,**e**,**h**,**k**,**n**), as well as the merged (**c**,**f**,**i**,**l**,**o**). Iba-1 + cells showed P2RY12 expression (arrowhead), except perivascular iba-1 + cells (**g**, arrow). P2RY12 expression was down-regulated in LG3d OHT (**d**–**f**). SLG3d OHT eyes showed only mild down-regulation of P2RY12 expression. NG, LG3d contralateral, and SLG3d contralateral eyes showed a similar P2RY12 expression. Abbreviations: Naïve age-matched control (NG), lasered group contralateral (LG3d contralateral), lasered group ocular hypertension (LG3d OHT), lasered group contralateral + saffron (SLG3d contralateral), lasered group ocular hypertension + saffron (SLG3d OHT).

In naïve animals, all Iba-1 + cells expressed P2RY12 except perivascular microglia and dendritic cells located in the juxtapapillary area and the peripheral retina. Most Iba-1 + cells in LG3d and SLG3d were P2RY12+. After laser induction on day 3 (LG3d OHT), P2RY12 expression in Iba-1 + cells was down-regulated in comparison with in NG, and treatment with saffron extract led to a smaller decrease in SLG3d OHT. The contralateral eyes of animals untreated (LG3d) or treated with saffron extract (SLG3d) expressed P2RY12 similarly to naïve eyes (Figure 10).

## 3. Discussion

This study was the first to demonstrate the neuroprotective and anti-inflammatory effects of saffron extract in an experimental mouse model of OHT. According to our results, saffron extract administration in OHT animals (i) prevented RGC death; (ii) decreased the morphological signs of microglial activation, and (iii) prevented the down-regulation of P2RY12 expression.

There has been only one previous study of saffron and glaucoma, which demonstrated that oral supplementation with saffron reduced the IOP in patients with primary open-angle glaucoma [45].

In the present work, we used a mouse model of unilateral, laser-induced OHT to study the possible protective effects of saffron extract in glaucomatous neuropathy. This is one of the experimental models available to study the mechanism by which IOP can cause retinal damage, and this model could be useful for testing neuroprotective substances to prevent ganglion cell death [3]. In unilateral, laser-induced OHT, a substantial increase in IOP was evident on the first day after laser photocoagulation of the limbal and episcleral veins. The IOP increase persisted until day 3 and then gradually returned to basal levels. The IOP values on day 7 were comparable to those of contralateral eyes [46,47]. In our study, saffron extract did not attenuate the increase in IOP caused by laser induction, as was observed in the study of patients with primary open-angle glaucoma [45]. This may have been because, in our mouse model, we increased the IOP via laser photocoagulation of the limbal and episcleral veins, which is different from the mechanism underlying the IOP increase in primary open-angle glaucoma.

Increases in IOP activate the microglia both in human [12] and in experimental models of OHT [6,7,11,13,48,49,50]. During the course of activation, microglia become proliferative and motile, migrating to damaged sites [51,52]. In addition, activated microglial cells undergo morphological changes, including an increase in soma size and process retraction [8,9,51]. Under a state of higher activation, microglia acquire an amoeboid morphology and act like macrophages [53]. Moreover, activated microglia can alter the expression of different receptors such as CX3C chemokine receptor 1 (CX3CR1), P2RY12, and cell surface transmembrane glycoprotein CD200 receptor 1 (CD200R1) [54].

In this experimental model, microglial activation was detected in OHT eyes at 24 h and 15 days after laser induction [6,7,10,11]. This microglial activation was caused by the IOP increase and was not simply an inflammatory reaction to laser treatment, as we demonstrated in a previous study using this OHT model [11]. In a previous work, we analyzed the time point after lasering at which microglial activation peaked, finding that it happened on day 3 after lasering [55]. This was the reason why we selected day 3 to analyze the possible anti-inflammatory effect of saffron extract. In the present study, we found that, in OHT eyes, microglial cells were activated 3 days after lasering, as demonstrated by an increase in soma size, process retraction, the presence of more vertical processes, and increases in the Iba1-RA and in the number of Iba-1 + cells. This increase was homogeneous in the different retinal zones (superior, inferior, nasal, and temporal), with no significant zone-specific differences observed. Most of these Iba-1 + cells were microglia, as we confirmed based on labeling with an anti-P2RY12 antibody. P2RY12 is expressed by microglia but not by dendritic cells, infiltrating macrophages or monocytes [56,57]. Upon activation, microglia express and release the inflammatory cytokines TNF-α, IL-1β, IL-6, and IL-12 [8,58,59,60], which, in turn, leads to the production of nitric oxide and reactive oxygen species (ROS), causing neuronal death [36,61,62]. Microglial activation has been associated with RGC death in both human [12] and OHT models [6,7,10,11]. Inhibiting microglial activation with high-dose radiation [15] or minocycline treatment [14] reduces RGC death in animal models of glaucoma. In addition, a deficiency in CX3CR1, which is expressed in the microglia, has been shown to increase microglial activation, inducing RGC loss, in an experimental mouse glaucoma model [63].

At 3 days after unilateral laser induction, animals treated with saffron extract (SLG OHT) presented (i) a significant decrease in the Iba-1 + cell number in all retinal layers analyzed (OS, OPL, IPL), (ii) a significant reduction in Iba1-RA in the NFL-GCL, (iii) a significant decrease in process retraction in the IPL, and (iv) a significant decrease in the cell body area in all studied layers. These results demonstrate that saffron extracts reduced the morphological signs of microglial activation induced by increased IOP. These anti-inflammatory effects of saffron could be related to its strong antioxidant and radical-scavenging properties [17]. Some investigators have postulated that saffron can regulate genes that control the release of proinflammatory cytokines from glial cells [35,42,64]. Other experimental studies have also shown the anti-inflammatory effect of saffron extracts. Crocins and crocetin have been shown to reverse LPS-induced activation and proliferation of microglia, as well as the production of nitric oxide, TNF-α, and IL-1β, by repressing NF-κB activity on cultured rat microglia, therefore exerting an anti-inflammatory effect [35]. In neuronally differentiated PC-12 cells, crocin blocked TNF-α-induced DNA fragmentation, suppressing cell death [65]. Pretreatment of cultured BV2 microglia with crocin reversed LPS-induced morphological changes, up-regulation of pro-inflammatory mediators (iNOS, Cox-2, IL-1 β, and TNF-α), and down-regulation of CX3CR1 expression [36]. In a mouse model of traumatic brain damage, crocin pretreatment markedly reduced the number of activated microglia and downregulated TNF-α and IL-1 β [66].

A reduction in microglial activation was also observed in contralateral eyes treated with saffron extract in comparison with untreated contralateral eyes. Microglial activation is constant in contralateral eyes after unilateral OHT induction, which was observed 3 days after lasering in the present study, and at other time-points after OHT induction in previous works by our group [6,7,10,11]. Microglial activation in contralateral eyes was not found to be related to RGC death, because the number of RGCs was similar between contralateral and naïve eyes. There are different theories to explain the microglial activation in the contralateral eye; these include principally hematic, through chiasm, and by the immune system [67]. Whatever the connection path between both eyes, the oral administration of saffron extract produces effects in OHT eyes and in their contralateral eyes, decreasing microglial activation.

In this study, we found that retinal microglial cells expressed P2RY12 receptors in naïve, LG3d, and SLG3d animals. P2RY12 receptors are selectively expressed by the microglia in the brain and are the critical purinergic receptor for the extension of microglial processes [68]. After damage, ATP, which is an important molecular chemoattractant mediating “find-me” signals, is released by neurons and activates P2RY12 receptors in microglial cells, triggering the extension of microglial processes toward the lesion zones [69]. In OHT eyes, we found a significant decrease in the RGC number, indicating neuronal damage. Immediately after damage, P2RY12 is up-regulated, and then it is down-regulated a few hours later, ultimately becoming undetectable in strongly activated microglial cells [70]. In accordance with this, in our study, we observed a decrease in P2RY12 expression to undetectable levels in Iba-1 + cells of OHT eyes at 3 days after laser induction, coinciding with an increase in the morphological signs of microglial activation. However, in animals treated with saffron extract, Iba-1 + cells of OHT eyes expressed detectable levels of P2RY12. This fact, together with the reduction in the morphological signs of microglial activation observed in these eyes, indicates that saffron extracts can dampen the activation of microglial cells. In contralateral eyes both treated and untreated with saffron, P2RY12 expression was similar to in naïve eyes. In addition, in contralateral eyes, the morphological signs of microglial activation were lower than in untreated OHT eyes. This could be due to the fact that the death of the RGCs does not occur in the contralateral eyes [3].

Thus, saffron extract can have a neuroprotective effect by reducing microglial activation and the associated production of neurotoxic molecules.

To assess the possible neuroprotective effect of the saffron extract, we did not select 3 days after lasering, the time point used to study the microglial activation. In this model, no data are available that indicate a decrease in the RGC number at 3 days after laser induction. However, previous studies have demonstrated that at 7 days after laser induction, there is a significant loss of RGCs in OHT eyes in the experimental model used in this study [3,71]. Thus, we chose this time-point to analyze the possible neuroprotective effect of saffron extract.

At 7 days after laser induction, as in previous studies [3,71], we found a significant decrease in the number of Brn3a + RGCs in OHT eyes, which was more evident in the superior zone. However, in the OHT eyes treated with saffron, this RGC loss did not occur, suggesting that saffron extract prevented RGC death induced by an increase in IOP at 7 days after laser induction. This neuroprotective effect of saffron or its main constituents has been found in other studies performed in different animal experimental models with retinal damage. Saffron extract treatments ameliorated the loss of photoreceptors in a model of autosomal dominant retinitis pigmentosa [40]. Saffron has been shown to protect retinal photoreceptors in a light damage model of photoreceptor degeneration in rats [72,73]. Crocetin reduces retinal cell death and apoptosis induced either by light exposure or by intravitreal administration of N-methyl-D-aspartate (NMDA) [18,19]. Finally, crocin and crocetin inhibit ischemia/reperfusion-induced retinal RGC death in mice and rats [38,74,75]. Thus, our work confirms the neuroprotective effect of saffron extract against retinal damage [76]. This protective effect could be related to its anti-inflammatory properties, as demonstrated in the present work, and to its antioxidant capacity. Crocetin scavenges ROS and stimulates the activity of endogenous antioxidant enzymes [38]. ROS and oxidative stress may cause apoptosis, and saffron extract can block neuronal death by apoptosis through down-regulation of Bax and caspase-3 expression [18].

In conclusion, the present study demonstrated that saffron extract standardized to 3% crocin content can produce anti-inflammatory and neuroprotective effects against the damage exerted by an increase in IOP in retinal tissue. Thus, saffron extract may help to protect RGCs from glaucomatous pathology.

## 4. Materials and Methods

### 4.1. Animals

Male Swiss albino mice, aged 12–16 weeks (40–45 g), were obtained from Charles River Laboratories (Barcelona, Spain) and kept in animal housing at the Faculty of Medicine of the Universidad Complutense de Madrid (Spain). Mice were given free access to water and a standardized diet and maintained on a 12 h light/dark cycle, with light intensity within the cages ranging from 9 to 24 lux, at a controlled temperature. The study protocol complied with the ethical guidelines endorsed by Spanish law and the Guidelines for Humane Endpoints for Animals Used in Biomedical Research. The study was approved by the Ethics Committee for Animal Research of Complutense University (Madrid, Spain) and the Directorate General of Agriculture and Food, Ministry of Economy and Employment of the Community of Madrid (approval ID number: ES280790000086, 1 April 2016). In addition, animal procedures followed institutional guidelines, European Union regulations for the use of animals in research, and the Association for Research in Vision and Ophthalmology (ARVO) statement for the use of animals in ophthalmic and vision research.

### 4.2. Experimental Groups

Animals were divided into the following groups. A naïve age-matched control group (NG, *n* = 6) did not undergo the experimental procedure, and one eye was studied in each animal. Lasered groups (LG) were exposed to a laser in one eye to induce unilateral OHT, after which the OHT and contralateral normotensive eyes were analyzed at 3 days (LG3d, *n* = 8) or 7 days (LG7d, *n* = 6). A saffron control group (SCG, *n* = 8) was treated like the NG group, except that they received saffron extract. Lasered + saffron groups (SLG) were treated like the LG groups, except that they received saffron extract and then were analyzed at 3 days (SLG3d, *n* = 8) or 7 days (SLG7d, *n* = 6).

### 4.3. Treatment with Saffron

Samples of hydrophilic stigma extracts of saffron (*Crocus sativus* L.) cultivated in Alborea (Albacete, Spain) were provided by Pharmactive Biotech Products S.L. (Madrid, Spain). The extracts, marketed under the brand name affron^®^EYE, were prepared using a proprietary method and analyzed by high performance liquid chromatography (HPLC) [77]. These extracts are typically standardized to contain more than 3% crocin isomers, and, apart from native saffron extract, they contain dextrin as a carrier. The affron^®^EYE batch in the present study contained 3.14% total crocins, all of which were in powder form and were stored in the dark until use in experiments. The extract used in this study was assayed to determine the ability of the naturally occurring crocins and their metabolic product, crocetin, to scavenge free radicals. In a 200 μg/mL extract, crocetin showed 3-fold higher 2,2-diphenyl-1-picryl-hydrazyl-hydrate (DPPH) scavenging activity than crocins [78].

The pretreatment period and the dose of saffron extract were decided based on previous findings [19,30,40,73,74,75]. Thus, saffron was administered during the 15 days before laser induction. In the SLG group, the saffron extract treatment was maintained after the laser procedure until animal sacrifice on day 3 (SLG3d group) or day 7 (SLG7d group). The dose of saffron extract was 60 mg/kg body weight per day, and it contained around 1.8 mg total crocins. To ensure that SCG and SLG mice received the correct dose of saffron extract, the extract was diluted in water and administered by gavage (0.01 mL/g body weight). In order to adjust the amount of saffron extract administered, each animal’s body weight was measured prior to saffron extract administration.

### 4.4. Anesthetics

Surgical procedures were performed under general anesthesia via intraperitoneal injection of a mixture of ketamine (75 mg/kg; Anesketin^®^, Dechra Veterinary Products SLU, Barcelona, Spain) and medetomidine (0.26 mg/kg; Medetor^®^, Virbac España S.A., Barcelona, Spain). During recovery from anesthesia, the mice were placed in their cages, and an ointment containing tobramycin (Tobrex^®^; Alcon, Barcelona, Spain) was applied to the cornea to prevent desiccation and infection. Additional measures were taken to minimize discomfort and pain after surgery. The animals were killed with an intraperitoneal overdose of pentobarbital (Dolethal Vetoquinol^®^, Especialidades Veterinarias, Alcobendas, Madrid, Spain). IOP was measured under inhalational anesthesia of 2% isoflurane in oxygen (ISOFLO Isoflurane 100% p/p, Zoetis SL, Alcobendas, Madrid, Spain).

### 4.5. Laser Treatment and Measurement of IOP

To induce OHT, the left eyes of the anesthetized mice were treated in a single session with a series of diode laser burns (Viridis Ophthalmic Photocoagulator-532 nm, Quantel Medical, Clermont-Ferrand, France) following previously described methods [46,47,79]. In brief, the laser beam was directly delivered without any lenses and was aimed at the limbal and episcleral veins. The laser spot size was 50–100 μm, and it was applied for a duration of 0.5 s with a power output of 0.3 W. Each eye received 55–76 burns. IOP was measured both in the laser-treated eye and in the contralateral eye using a rebound tonometer (Tono-Lab, Tiolat, OY, Helsinki, Finland) [3]. IOP was measured prior to saffron administration in SCG and SLG, and prior to laser induction in LG and SLG. In LG3d and SLG3d, IOP was measured on days 1, 2, and 3 after laser induction; in LG7d and SLG7d, IOP was measured on days 1, 2, 3, 5, and 7 after lasering. In the NG and SCG, IOP was measured just before sacrifice. To minimize IOP variations due to the circadian rhythm in albino Swiss mice or spontaneous increases, we measured IOP around the same time, preferentially at 9 a.m., and directly after inhalational anesthesia. Six consecutive measurements were made in each eye and the average was calculated [3].

### 4.6. Immunohistochemistry

Mice were deeply anesthetized, as mentioned above, and then perfused transcardially through the ascending aorta, first with saline and then with 4% paraformaldehyde in 0.1 M phosphate buffer (PB, pH 7.4). The orientation of each eye was carefully maintained with a suture placed on the superior pole immediately after deep anesthesia and before perfusion fixation. During eye dissection, the insertion of the rectus muscle and the nasal caruncle were used as additional landmarks. Eyes were post-fixed for 2 h in the same fixative and kept in sterile 0.1 M PB. The retinas were then dissected, the vitreous humor was removed using atraumatic clamps and Westcott scissors (vitrectomy), and whole-retina mounts were prepared [80].

Different primary antibodies were used for the immunohistochemistry analysis. To evaluate microglial activation through cell morphology, we used an antibody against ionized calcium binding adaptor molecule 1 (Iba-1), which revealed the morphological features of microglia [81]. In order to differentiate resident retinal microglia from macrophages or microglia derived from infiltrated monocytes, as well as differentiate resting microglia from activated microglia, we used an antibody against the P2 purinergic receptor P2RY12 [56,70]. To quantify the RGC number, we used an antibody against brain-specific homeobox/POU domain protein 3A (Brn3a), a marker localized in the RGC nuclei that is down-regulated before cell death [3].

Retina whole mounts were immunostained as previously described [6,7,10,11]. In brief, retinas of NG (*n* = 6), LG3d OHT (*n* = 6), LG3d contralateral (*n* = 6), SCG (*n* = 6), SLG3d OHT (*n* = 6), and SLG3d contralateral (*n* = 6) eyes were immunostained with rabbit anti-Iba-1 (1:600; Wako, Osaka, Japan), followed by a secondary antibody, donkey anti-rabbit IgG Alexa Fluor 594 (1:800; Invitrogen, Paisley, UK).

Retinas of NG (*n* = 2), LG3d OHT (*n* = 2), LG3d contralateral (*n* = 2), SCG (*n* = 2), SLG3d OHT (*n* = 2), and SLG3d contralateral (*n* = 2) eyes were double-immunostained with rat anti-P2RY12 (1:100; Biolegend, San Diego, USA) and rabbit anti-Iba-1 (1:600; Wako, Osaka, Japan), followed by the corresponding secondary antibodies: goat anti-rat IgG Alexa Fluor 488 (1:150; Invitrogen, Paisley, UK) and donkey anti-rabbit IgG Alexa Fluor 594 (1:800; Invitrogen, Paisley, UK).

Retinas of NG (*n* = 6), LG7d OHT (*n* = 6), LG7d contralateral (*n* = 6), SCG (*n* = 6), SLG7d OHT (*n* = 6), and SLG7d contralateral (*n* = 6) eyes were double-immunostained with mouse anti-Brn3a (1:300; MAB-1585; Millipore Corp., Billerica, MA, USA) and rabbit anti-Iba-1 (1:600; Wako, Osaka, Japan), followed by goat anti-mouse IgG Alexa Fluor 488 (1:800; Invitrogen, Paisley, UK) and donkey anti-rabbit IgG Alexa Fluor 594 (1:800; Invitrogen, Paisley, UK).

In order to verify that secondary antibodies reacted only with their respective primary antibodies, three negative controls were included. In one control, the primary antibody was omitted. In the second one, the secondary antibody was omitted. In the third one, both primary and secondary antibodies were omitted and tissue samples were incubated only in the corresponding diluent solutions, which also allowed us to evaluate the amount of endogenous fluorescence applied to the observed label [82].

Retinas were analyzed and photographed with an ApoTome 2-slider module (Carl Zeiss, Oberkochen, Germany) and a high-resolution digital camera Axio CAM 503 Mono (Carl Zeiss) coupled to a fluorescence microscope (Zeiss Axio Imager M.2, Carl Zeiss), as previously described [10]. The microscope was equipped with a Zeiss filter set 64 for Alexa Fluor 594 and a Zeiss filter set 10 for Alexa Fluor 488. The ApoTome device enables conventional microscopy to create optical sections through the specimen, and thereby improves the contrast and resolution along the optical axis. Each whole-retina mount was analyzed along the entire *x*-, *y*-, and *z*-axes using a motorized stage. Cellular components in the same x–z plane were considered to lie in the same focal plane. *Z*-stacks were analyzed using ZEN2 software (Carl Zeiss, Oberkochen, Germany). Figures were prepared using Adobe Photoshop CS3 Extended 10.0 (Adobe Systems, San Jose, CA, USA).

### 4.7. Quantitative Retina Analysis

#### 4.7.1. Iba-1 + Cells

To determine the effect of OHT on Iba-1 + cells as well as the possible anti-inflammatory saffron extract effect, we quantified (a) the number of Iba-1 + cells in the outer segment (OS), the outer plexiform layer (OPL), and the inner plexiform layer (IPL); (b) the area of the retina occupied by Iba-1 + cells (Iba1-RA) in the nerve fiber layer-ganglion cell layer (NFL-GCL); (c) the arbor area of the Iba-1 + cells in the OPL and IPL; (d) the number of microglial vertical processes connecting the OPL and OS; and (e) the cell body area of Iba-1 + cells in the OPL, IPL, and NFL-GCL. All quantifications were performed in a double-blind fashion. The OS, OPL, IPL, and NFL-GCL were identified based on differences in microglial morphology. The borders between retinal layers were recognized based on the weak auto fluorescence emitted by somata in the nuclear layers, which was detected using the Zeiss filter set 10 for Alexa Fluor 488.

Quantification was performed in NG (*n* = 6), LG3d OHT (*n* = 6), LG3d contralateral (*n* = 6), SCG (*n* = 6), SLG3d OHT (*n* = 6), and SLG3d contralateral (*n* = 6) eyes, as previously described by our group [7,83]. For the analysis of the number of Iba-1 + cells, equivalent areas of the retina were consistently selected and photographed for each retinal whole mount in both vertical and horizontal meridians that crossed the optic nerve (including the superior, inferior, nasal, and temporal zones). All fields subsequently analyzed were contiguous to ensure that no portion of the retinal whole mount was omitted or duplicated. Each meridian was analyzed using the motorized stage of the microscope to scan its entire length along the *x*–*y* axis, giving an approximate total of 550 fields evaluated [7,83]. These areas were photographed at a magnification of 20×, giving an area of 0.1502 mm^2^ per field. Additionally, to quantify the number of Iba-1 + cells in the OPL, IPL, and NFL-GCL, we analyzed the entire preparation along the *z*-axis in depth for every 2 μm section.

The quantification method used depended on the cell number and cell distribution characteristics in each retinal layer. In the IPL and OPL, Iba-1 + cells formed a cellular network; they were distributed throughout the retina in a non-overlapping mosaic arrangement, allowing individual identification and automatic cell counting [83,84]. In the NFL-GCL and in the OS, the distribution of Iba-1 + cells was not regular and did not allow automatic cell counting; thus, in the NFL-GCL, we quantified the Iba1-RA [83,84], and in the OS, we performed manual quantification.

##### Number of Iba-1 + Cells in the OS

For Iba-1 + cell quantification in this layer, we used the “Interactive” manual counting tool in ZEN2 software (Carl Zeiss) in association with the ApoTome device coupled to the fluorescence microscope.

##### Number of Iba-1 + Cells in the OPL, IPL, and NFL-GCL

Iba-1 + cells were counted using a rapid, reliable automatic segmentation and distance-control algorithm developed by our group in MATLAB (MathWorks, Natick, MS, USA) [84]. This algorithm quantified Iba-1 + cells numbers in the OPL and IPL as well as Iba1-RA cells in the NFL-GCL. In brief, Iba1 + cells were counted as follows: selected images were averaged as a *z*-stack, giving rise to a *z*-projection, which was processed in two ways in order to preserve only the most intense part of the Iba-1 + cells (normally the cell body). This characteristic was used to identify and quantify Iba-1 + cells [83,84]. In the first step, the image was normalized to the pixel with the peak value in the image, such that image values fell within a range from 0 to 1. Next, a threshold was imposed and all values <0.2 were set to 0, while the remaining values were retained. The remaining image was segmented, and the center of mass of each segment was determined in order to identify the presence or absence of cells. To prevent multiple counting of the same cell appearing in adjacent segments, we specified that different cells must lie at a minimum distance from each other. All points closer to each other than this minimum distance were considered to belong to the same cell and were counted only once.

In the NFL-GCL, in each selected image, we quantified Iba1-RA cells using a threshold tool in MATLAB [83,84]. The threshold defined the range of grayscale values for pixels containing objects of interest in order to differentiate them from other parts of the image. We modified the threshold value and then used the “count NFL” algorithm to determine the percentage of retinal area labeled by anti-Iba1.

##### Arbor Area of Iba-1 + Cells in the OPL and IPL

In each plexiform layer, we chose four equivalent retinal areas among those used for Iba-1 + cell counting in the retinal whole mounts. To ensure the reliability of our results, we selected retinal areas located at specific distances from the optic disc in the different retinal quadrants as follows: in the superior retina, the retinal area closest to the optic disc; in the inferior retina, the area at two levels of eccentricity from the disc; in the nasal retina, the area at three levels of eccentricity; and in the temporal retina, the area at four levels of eccentricity. In the selected 20× photomicrographs, a polygon was drawn manually by connecting the distal-most tips of the Iba-1 + cells processes using the “Interactive Measurement” tool in ZEN2 software (Carl Zeiss), together with the ApoTome device coupled to the fluorescence microscope. A computer-assisted morphometric algorithm then quantified the arbor area of the Iba-1 + cells [7].

##### Number of Vertical Processes of Iba-1 + Cells Connecting the OPL and OS

In the four retinal areas selected for arbor area quantification, we made photomicrographs at 20 × of Iba-1 + spots observed in the plane between the OPL and OS. These spots corresponded to the vertical processes of Iba-1 + cells connecting the OPL and OS. We quantified these spots using the manual counting tool in ZEN2 software.

##### Cell Body Area of Iba-1 + Cells in the OPL, IPL, and NFL-GCL

We took photomicrographs at 20× magnification in the OPL, IPL, and NFL-GCL in the same retinal areas as those used for the quantification of the arbor area and the vertical processes. We manually drew the contour of the Iba-1 + somata to determine their area, using the “Interactive measurement tool” in ZEN2 software.

#### 4.7.2. RGCs

To determine the effect of OHT on RGCs and the possible neuroprotective effects of saffron, we quantified the number of Brn3a + RGCs using a double-blind procedure. Quantification was performed in NG (*n* = 6), LG7d OHT (*n* = 6), LG7d contralateral (*n* = 6), SCG (*n* = 6), SLG7d OHT (*n* = 6), and SLG7d contralateral (*n* = 6) eyes. To count the Brn3a + RGCs, equivalent areas of the retina in the RGC layer were consistently selected and photographed for each retinal whole mount in both vertical and horizontal meridians that crossed the optic nerve (including superior, inferior, nasal, and temporal zones). All subsequently analyzed fields were contiguous to ensure that no portion of the retinal whole mount was omitted or duplicated. Each meridian was analyzed using the motorized stage of the microscope to scan its entire length along the *x*–*y* axis, giving an approximate total of 550 fields evaluated [7,83]. These areas were photographed at a magnification of 20×, giving an area of 0.1502 mm^2^ per field.

For Brn3a + RGCs quantification, we applied the same algorithm developed in MATLAB for automatic counting of Iba-1 + cells [83,84]. In this case, we specified the minimum distance between RGCs to allow counting only once.

### 4.8. Statistical Analysis

Data were analyzed statistically using SPSS version 22 (IBM, Chicago, IL, USA) and reported as the mean ± standard deviation (SD). Differences among NG, SCG, LG3d, SLG3d, LG7d, and SLG7d were assessed for significance using the Mann–Whitney U test (in the case of unpaired data) or the Wilcoxon W test (in the case of differences between OHT and contralateral eyes). The compared parameters were (i) the IOP; (ii) the Iba-1 + cell number in the OS, OPL, and IPL; (iii) Iba1-RA in the NFL-GCL; (iv) the arbor area of Iba-1 + cells in the OPL and IPL; (v) the number of microglial vertical processes connecting the OPL and OS; (vi) the cell body area of Iba-1 + cells in the OPL, IPL, and NFL-GCL; and (vii) the Brn3a + RGCs number. Differences were considered statistically significant when *p* < 0.05.

Differences in the following parameters among the superior, inferior, nasal, and temporal retinal zones in OHT eyes were assessed for significance using the ANOVA test with Bonferroni correction: (i) number of Iba-1 + cells in the OS, OPL, and IPL in the LG3d OHT and SLG3d OHT; (ii) Iba1-RA in the NFL-GCL in the LG3d OHT and SLG3d OHT; and (iii) the number of Brn3a + RGCs in the LG7d OHT and SLG7d OHT. Differences were considered significant when *p* < 0.05.
